# Gaitmap—An Open Ecosystem for IMU-Based Human Gait Analysis and Algorithm Benchmarking

**DOI:** 10.1109/OJEMB.2024.3356791

**Published:** 2024-01-22

**Authors:** Arne Küderle, Martin Ullrich, Nils Roth, Malte Ollenschläger, Alzhraa A. Ibrahim, Hamid Moradi, Robert Richer, Ann-Kristin Seifer, Matthias Zürl, Raul C. Sîmpetru, Liv Herzer, Dominik Prossel, Felix Kluge, Bjoern M. Eskofier

**Affiliations:** Machine Learning and Data Analytics LabFriedrich-Alexander Universität Erlangen-Nürnberg (FAU) 9171 91054 Erlangen Germany; Machine Learning and Data Analytics LabFriedrich-Alexander Universität Erlangen-Nürnberg (FAU) 9171 91054 Erlangen Germany; Department of Molecular NeurologyFAU Erlangen 9171 91054 Erlangen Germany; Computer Science Department, Faculty of Computers and InformationAssiut University Assiut Governorate 71515 Egypt

**Keywords:** Accelerometer, walking, biomarker, biomechanics, movement analysis

## Abstract

*Goal:* Gait analysis using inertial measurement units (IMUs) has emerged as a promising method for monitoring movement disorders. However, the lack of public data and easy-to-use open-source algorithms hinders method comparison and clinical application development. To address these challenges, this publication introduces the gaitmap ecosystem, a comprehensive set of open source Python packages for gait analysis using foot-worn IMUs. *Methods:* This initial release includes over 20 state-of-the-art algorithms, enables easy access to seven datasets, and provides eight benchmark challenges with reference implementations. Together with its extensive documentation and tooling, it enables rapid development and validation of new algorithm and provides a foundation for novel clinical applications. *Conclusion:* The published software projects represent a pioneering effort to establish an open-source ecosystem for IMU-based gait analysis. We believe that this work can democratize the access to high-quality algorithm and serve as a driver for open and reproducible research in the field of human gait analysis and beyond.

## I. Introduction

In Recent years, there has been a significant increase in research activity and advancements within the field of human gait analysis. In particular, the use of small and lightweight inertial measurement units (IMUs) has enabled the collection of gait data in a variety of settings, allowing for a wide range of applications in the fields of sports, rehabilitation, and health monitoring [1], [2], [3], [4]. Gait analysis based on IMUs often requires complex multistep algorithms to extract meaningful parameters from the recorded raw data. Multiple approaches for the various steps (e.g. stride segmentation or stride length estimation) of the analytic pipelines have been proposed in the literature [5], [6].

Unfortunately, many of the proposed algorithms are only evaluated on single datasets and are not compared to other approaches. Further, comparison metrics are often inconsistent, making it difficult to compare results across studies. To overcome that, multiple benchmark studies were performed to objectively compare algorithms for specific applications [7], [8], [9], [10], [11], [12], [13], [14], [15]. However, even the most comprehensive studies only compare a small subset of the reported algorithms. One of the core challenges is that authors need to implement most of the algorithms from scratch, as high quality source code is usually not publicly available. In combination with the lack of high quality public datasets, no conclusive algorithm ranking exists for the majority of IMU-based gait analysis tasks.

The lack of easy to use open source gait analysis algorithms leads to duplicated work across the field and a higher likelihood of implementation errors affecting published results. Further, it increases the barrier of entry into the field and prevents applications and research based on gait parameters without detailed understanding of the fundamental algorithms or access to proprietary implementations.

Mandates from public funding agencies [16], and government programs [17] might motivate some authors to share their work more openly. However, in most cases intrinsic motivation of individual researchers is the primary driving factor. Given this lack of direct incentive, the required effort often appears to be disproportionately large [18], [19], [20], [21]. Unfortunately, attempts to support researchers with the associated technical challenges are rare.

Still, notable exceptions exist in the field on IMU-based gait analysis, who published their code publicly without restrictions [14], [15], [22], [23], [24], [25], [26], [27], [28], [29]. But, the scope of most of these code publications is limited to providing the used algorithms “as-is”, with reproducibility and not reusability as the primary goal. Only a handful of projects attempt to build reusable software libraries. The most widely used is *OpenSim*, which implements a wide range of biomechanical simulation tools, including gait analysis using inverse-kinematics [30]. This approach focuses on full reconstruction of lower-limb mechanics using multi-sensor setups typically used in a laboratory setting. The *gaitpy* package [22] and its successor *scikit-digital-health* [31] provide an algorithm pipeline for hip-worn sensors focusing on fundamental spatial-temporal gait parameters. However, to the best of our knowledge, these two packages have not been widely adopted. This leaves a considerable gap in the availability of easy to use and high quality software libraries for IMU-based gait analysis, in particular for “few-node” setups applicable for long-term unsupervised monitoring.

Other scientific fields show that investing in open and well maintained tooling can have a significant impact on the scientific progress. As a prime example, *scikit-learn* [32] has revolutionized the field of machine learning by vastly simplifying the process of working with common algorithms. Its algorithm interface has become the de-facto standard for machine learning in Python, enhancing code standardization, correctness, and facilitating reuse and extension of research code by others. In addition, projects like *Kaggle*^1^^1^https://www.kaggle.com and *Papers with Code*^2^^2^https://paperswithcode.com provide accessible platforms for open benchmarking of machine learning methods. This makes it easier to identify and reuse the best available methods and perform meaningful comparisons of new methods.

Given the clear benefits of easy to use computational tools, we believe that a standardized and open ecosystem for IMU-based gait analysis would have a significant positive impact on the field. It would not only support the development and validation of algorithms, but also make state-of-the-art methods accessible for clinical research and support the development of end-user products. With this publication and its accompanying software libraries, we hope to provide a starting-point for such an ecosystem with a focus on foot-worn IMUs. Specifically, we present the following contributions:

1) The *gaitmap*^3^^3^https://github.com/mad-lab-fau/gaitmap library, which provides easy to use Python implementations of more than 20 algorithms for foot-worn IMUs based on 17 papers and a wide array of utility functions for working with IMU data.
2) The *gaitmap-datasets*^4^^4^https://github.com/mad-lab-fau/gaitmap-datasets library, which provides easy access to existing public datasets.
3) An extensible platform (*gaitmap-challenges*, *gaitmap-bench*) to create fully reproducible benchmarks for gait analysis algorithms.^5^^5^https://github.com/mad-lab-fau/gaitmap-bench Currently, it provides eight concrete benchmark challenges with reference implementations.

## II. Materials and Methods

### A. Overview Over the Gaitmap Ecosystem

**Fig. 1.**
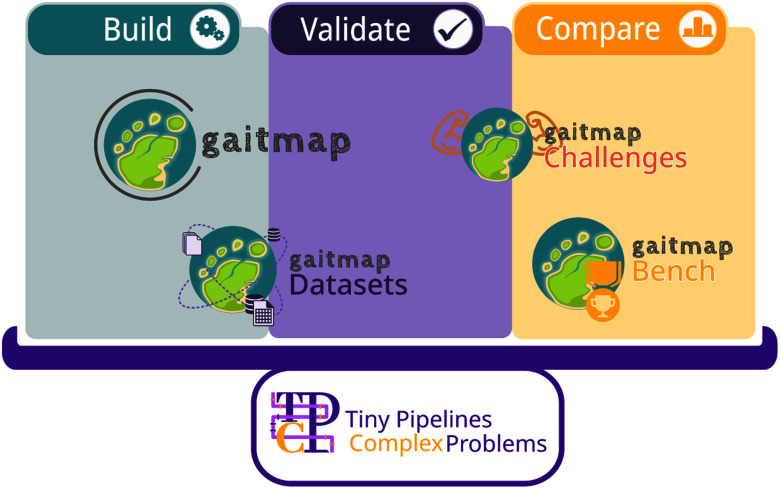
Overview of the *gaitmap* ecosystem, showing the four core packages (*gaitmap*, *gaitmap-datasets*, *gaitmap-challenges*, *gaitmap-bench*). These packages can support researchers in building gait analysis pipelines, validating pipelines and algorithms, and finally publishing and comparing reproducible benchmark results. The domain-agnostic *tpcp* package is used as the foundation for all other packages.

The presented ecosystem consists of four core packages that all build on top the domain-agnostic *tpcp* package [33] (Fig. 1). All packages are implemented in Python and follow modern best-practices in regard to structure and software-development and are build on top of widely used scientific Python packages (NumPy [33], SciPy [34], pandas [35], scikit-learn [32], Optuna [36], pomegranate [37], and tslearn [38]). The source of all *gaitmap* packages is published via Github (github.com/mad-lab-fau/gaitmap) and their extensive documentation is hosted on ReadTheDocs (gaitmap.readthedocs.io).

**Fig. 2.**
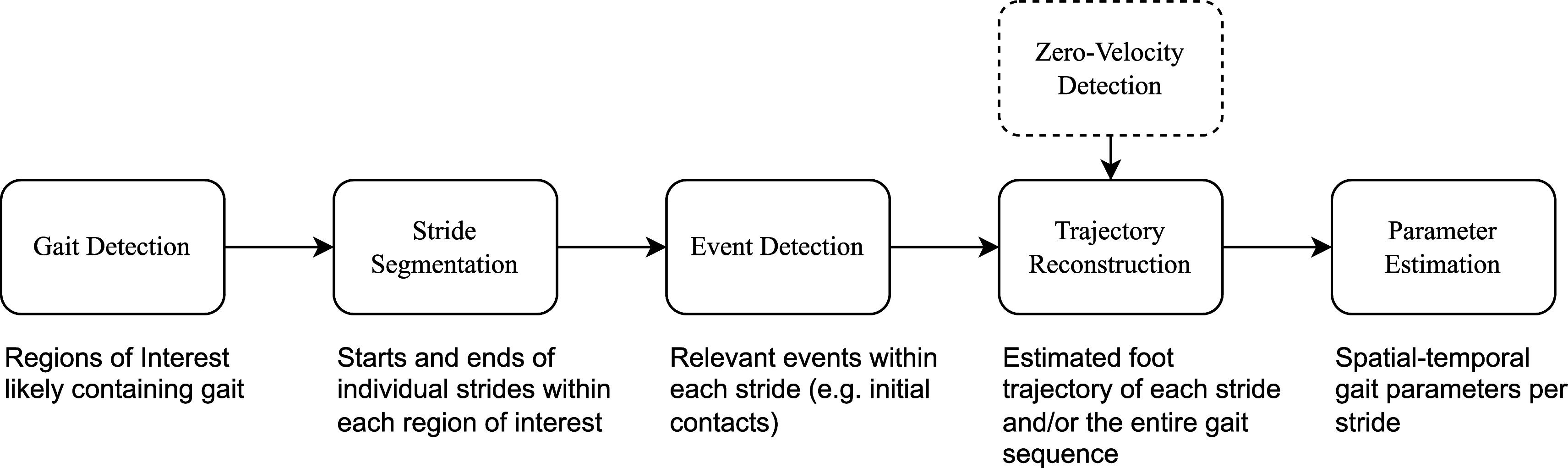
Overview of the main gait analysis pipeline that can be built using the *gaitmap* package. The pipeline is inspired by Rampp et al. [39] with *Gait Detection* as an additional step. After the gait detection, each algorithm is expected to work on one of the detected regions of interests and has access to the results of the previous steps. The text under each box provides a short description of the output of each step.

The *gaitmap* package provides core algorithms for gait analysis using foot-worn IMUs aligned along a pipeline inspired by Rampp et al. [39] (Fig. 2). For each of the steps in the pipeline *gaitmap* provides a number of interchangeable algorithms (Table I). Each algorithm is implemented as a Python class, providing attributes to configure the algorithm parameters (Appendix 1, (Supplementary material)). The algorithms can be used independently or combined into a pipeline to run an end-to-end analysis. For each step users can substitute algorithms with their own custom implementations. Beyond the core algorithms, the package provides a number of utility methods to preprocess data, simplify the development of custom algorithms, and to evaluate the output of algorithms against ground truth references.

**TABLE I:** Overview of the Core Algorithms Provided in the *Gaitmap* Package

**Processing Step**	**Algorithm**	**Description**
Preprocessing	align_dataset_to_gravity	Alignment of IMU signal with gravity.
	align_heading_of_sensors	Align the heading to the movement direction using PCA on the gyroscope signal and an integration of the acceleration signal (unpublished, inspired by [40]).
Gait Detection	UllrichGaitSequenceDetection	Detection of gait using harmonic frequencies from raw IMU signals [41]*.
Stride Segmentation	BarthDtw	Template matching using dynamic time warping (DTW) modified based on [42]*.
	ConstrainedBarthDtw	BarthDtw with additional local warping constraints. Inspired by local weightings explained in [43].
	HmmStrideSegmentation	Multi-activity Hidden-Markov-Model (HMM) based on [44]* implemented using [37].
Event Detection	RamppEventDetection	Initial and terminal foot contact detection based on signal features [39]*.
	FilteredRamppEventDetection	RamppEventDetection with additional lowpass filtering for specific calculation steps.
	HerzerEventDetection	Initial and terminal foot contact based on signal features tuned for stair walking [45]*. Combines concepts from [39]* and [46].
Zero-Velocity Detection	AredZuptDetector	Window based thresholding on gyroscope norm to detect static regions [47].
	NormZuptDetector	Window based thresholding to detect static regions. Generalized version of ARED from [47].
	ShoeZuptDetector	Window based thresholding on combined gyroscope and accelerometer norm to detect static regions [47].
Trajectory Reconstruction	RtsKalman	Zero-Velocity aided Error Tracking Kalman Filter with smoothing to estimate the foot trajectory from IMU signals. Based on [48], [49] with implementation details from [50].
	MadwickRtsKalman	Variation of the RtsKalman method using the Madgwick algorithms for orientation estimation (unpublished, based on [24], [48], [49], [50]).
	SimpleGyroIntegration	Orientation estimation based on integration of the gyroscope signal.
	MadgwickAHRS	Sensor fusion algorithm for orientation estimation [24].
	ForwardBackwardIntegration	Position estimation using double-integration of the acceleration with dedrifting through forward-backwards integration [51] and [9]*.
	PieceWiseLinearDedriftedIntegration	Position estimation using double-integration of the acceleration with linear drift model (unpublished, inspired by [52]).
Parameter Estimation	TemporalParameterCalculation	Calculation of temporal parameters (e.g. stride time) based on the detected events.
	SpatialParameterCalculation	Calculation of spatial parameters (e.g. stride length) based on the calculated trajectory [39], [53]*.

The Name in the *Algorithm* Column Corresponds to the class/function Name Within the Package. The Last Column Lists the Publications That the Algorithm is Based On. Algorithms Marked With an Asterisk (*) are Publications That Were Published by the Authors of This Manuscript Directly or the Associated Research Group. Besides the Core Algorithms, the Package Provides a Number of Helper Methods, Which are Not Listed Here. For a Full List See the Official Documentation Page (gaitmap.readthedocs.io). The Categories are Listed in the Order They are Typically Used in a Gait Analysis Pipeline.

These evaluation methods are used in the *gaitmap-challenges* package, which provides a standardized framework to run and evaluate gait analysis pipelines on specific challenges. Each benchmark challenge is either based on a train-test split or a cross-validation approach, allowing for proper comparison of machine learning and traditional algorithms alike. These challenges can be run fully locally on any *gaitmap*-compatible implementation to enable quick validation and standardized comparison of algorithms. All predefined challenges use *gaitmap-datasets* as the interface to load and standardize the publicly available datasets they are based on.

Finally, *gaitmap-bench* presents a collection of reproducible scripts implementing various challenges of the *gaitmap-challenges* package for a number of different algorithms. The results of these scripts are published on the *gaitmap-bench* website,^6^^6^https://gaitmap-bench.readthedocs.io which provides a visual overview of the results and further information about the respective challenges. New scripts and results for other public algorithms can be submitted by anyone via GitHub.

### B. Data Standardization

To ensure that algorithms can be compared across multiple datasets, we propose standardization guidelines for the input and output datatypes. The two main aspects of this standardization concern the used units and coordinate systems. Regarding the units, we suggest using the International System of Units (SI) wherever possible (Table II). We only make an exception for angular values, where we suggest using degrees instead of radians, as they are more comprehensible. For the orientation of the sensor or the body/foot, we use a *quaternion* representation.

**TABLE II:** Overview of the Units Used in the *Gaitmap* Package

**Metric**	**Unit**
Distance	Meter $(\mathrm{m})$
Time	Seconds $(\mathrm{s})$
Acceleration	Meter per second squared $(\mathrm{m/}\mathrm{s}^{2})$
Angular Velocity	Degrees per second $(\deg /\mathrm{s})$
Orientation	Unit quaternion in $(x, y, z, w)$ order

**Fig. 3.**
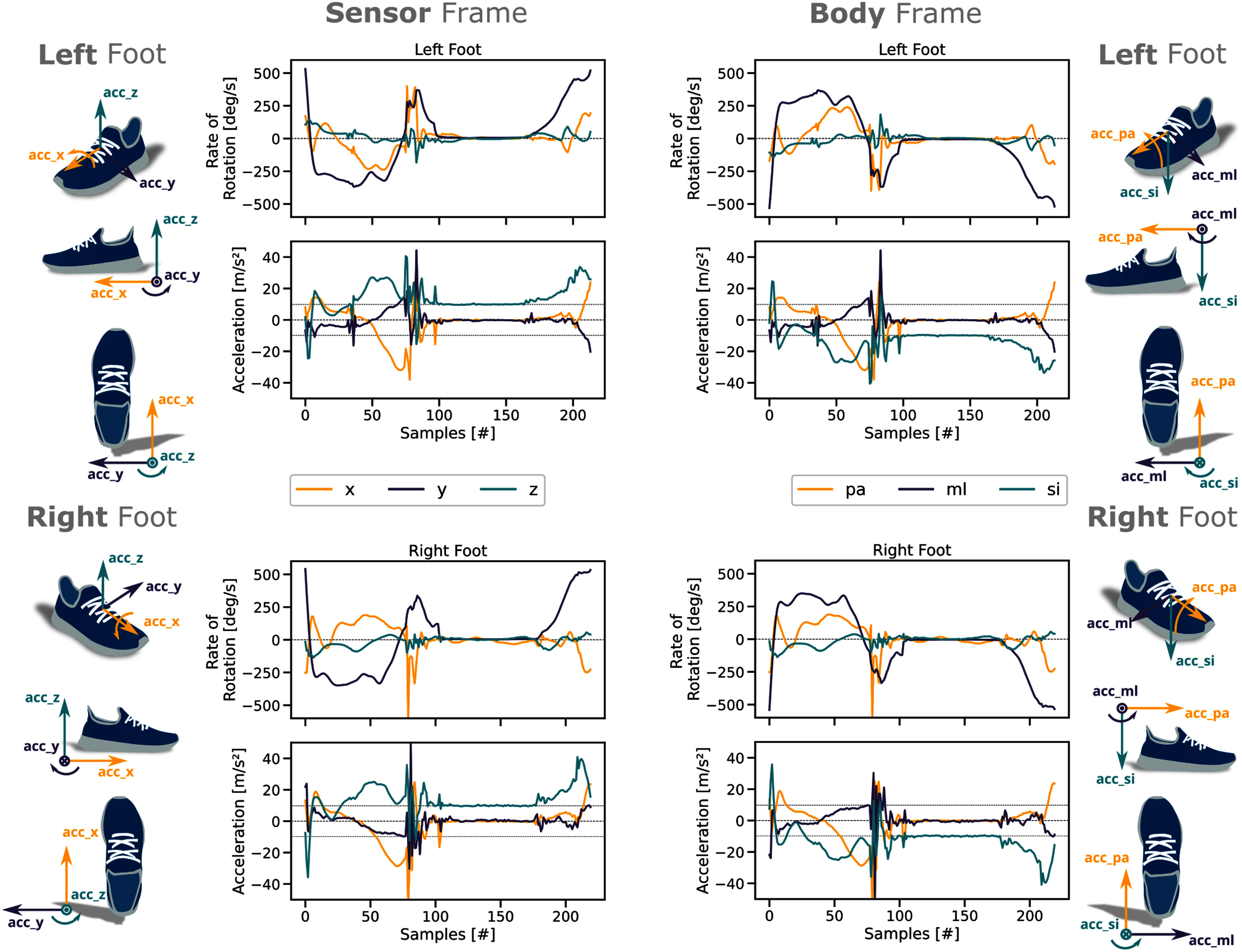
Illustration of the sensor and the body frame with example signals from an individual stride (toe-off to toe-off) of a healthy participant recorded with a foot-worn IMU sensor at 204.8 Hz. The direction of the arrows indicate the movement/rotation direction resulting in a positive sensor reading. The coordinate system for the sensor frame (left) is defined by the three axes $x$, $y$, and $z$ of the sensor. The coordinate system for the body frame (right) is defined by the main body axes $ml$ (medio-lateral), $pa$ (posterior-anterior), and $si$ (superior-inferior) of the foot.

Regarding coordinate systems, the optimal choice will depend on the sensor position. For foot/leg-mounted IMUs we propose to differentiate between three coordinate systems: (1) the sensor frame, (2) the body frame, and (3) the world (or global) frame. The sensor frame is created by the physical axes of the IMU sensor. We expect that the $x$ axis is pointing roughly forward, the $y$ axis to the left, and the $z$ axis upwards relative to the body of a participant. The body frame is defined by the three main body axes, namely $ml$ (medial to lateral), $pa$ (posterior to anterior), and $si$ (superior to inferior) (Fig. 3). Directions of rotations are defined based on the International Society of Biomechanics (ISB) recommendations for the ankle joint [54]. The resulting body frame coordinate systems of the left and right foot are mirror images of each other. In result, the same signals can be expected for the same anatomical movements independent of the limb. This is beneficial when performing any form of event detection on the signal. Finally, the world frame is used to report the global displacement or change of orientation of the sensor or body over a period of time.

### C. Benchmarking

Validating and benchmarking is essential during algorithm development. The presented ecosystem makes this process simple and standardized by (1) providing scoring functions specific for gait parameters to compare algorithm results against a ground truth, (2) providing primitives to perform parameter optimization and cross-validation, and (3) providing a set of challenges that allow benchmarking algorithms on a specific dataset with a single method call (Appendix 1, (Supplementary material)). Together, this decreases the chance of implementation errors and reduces the workload for comparing algorithms across multiple datasets.

At the time of writing, eight of these challenges are provided (Table III) and reference implementations and results for all *gaitmap* algorithms exist as part of the *gaitmap-bench* repository. In this manuscript, we present two of these challenges as an example in more detail. Further information and detailed results can be found on the *gaitmap-bench* website^7^^7^https://gaitmap-bench.readthedocs.io and Appendix 2, (Supplementary material).

**TABLE III:** Overview of the Benchmark Challenges Currently Implemented in the *Gaitmap-Challenges* Package

**Processing Step**	**Dataset**	**Description**
Stride Segmentation	EgaitSegmentationValidation2014 [42]	Algorithms need to identify individual strides based on the definition by Barth et al. [42] during supervised 4 × 10 m gait tests and unstructured simulated free-living activities. The sensors are mounted laterally at the shoes.
	EgaitSegmentationValidation2014 [42] (relabeled)	**Same as the previous challenge, but in addition to straight strides the algorithms also need to identify strides during stair walking and turns.**
	SensorPositionComparison2019 [55]	Algorithms need to identify individual strides based on the definition by Barth et al. [42] during various walking tasks in the laboratory. Two synchronized IMU sensors are mounted on the instep of the shoe.
Spatial Parameter	EgaitAdidas2014 [53]	Algorithms need to estimate the stride length of strides performed at different speeds within the capture volume of a MoCap system. Two sensors are mounted laterally on the shoe and predefined stride borders and events are provided.
	EgaitParameterValidation2013 [39]	Algorithms need to estimate the stride length of strides performed by geriatric patients while walking over a GAITRite carpet. Two sensors are mounted laterally on the shoe and predefined stride borders and events are provided.
	SensorPositionComparison2019 [55]	Algorithms need to estimate the stride length of strides performed at different speeds within the capture volume of a MoCap system. Two synchronized IMU sensors are mounted on the instep of the shoe and predefined stride borders and events are provided.
Full Pipeline	SensorPositionComparison2019 [55]	Algorithms need to estimate the mean and standard deviation of the stride length, gait velocity, and stride time of each of the performed gait tests. Only the raw IMU data of the two sensors mounted on the insteps of the shoes is provided.
	KlugeParameterValidation2017 [56]	**Algorithms need to estimate the mean and standard deviation of the stride length, gait velocity, and stride time of each of the performed 4 × 10 m tests. Only the raw IMU data of the two sensors mounted laterally on the shoes is provided.**

The Two Challenges With Descriptions Highlighted in Bold are Presented in More Detail in This Manuscript. The *Dataset* Column Provides the Class Name of the Dataset Within the *Gaitmap-Datasets* Package.

#### 1) Example 1 – Stride Segmentation

We use the dataset by Barth et al. [42] (30 participants performing a 4 × 10 m walk and 15 participants performing a 4 min free-walk) to benchmark the extraction of individual strides from a continuous signal of foot-worn IMUs. We consider a stride as detected (“true-positive”), if both the start and end label are within 30 ms of the hand-labeled ground truth. Based on this output we calculated the precision, recall, and F1-score. The validation is performed within a 5-fold cross-validation to allow for parameter optimization on an independent training set.

On this challenge we compare the three stride segmentation algorithms implemented in *gaitmap* (*DTW*, *Constrained-DTW*, *HMM*). We tested each algorithm once with its default parameters and once with parameters automatically optimized for the dataset. For the *HMM* this involved retraining the underlying model from scratch. See Appendix 2, (Supplementary material) for more details.

#### 2) Example 2 – Full Pipeline Validation

To evaluate the representative performance of entire gait analysis pipelines, we compare aggregated parameters over an entire gait test. These types of comparisons represent more closely the error ranges expected during actual usage of a gait analysis system and, hence, are an important addition to detailed validation of individual algorithms [57].

The specific challenge we present in this manuscript uses the dataset recorded by Kluge et al. [56] containing recordings of 4 × 10 m walk tests at different speeds from 11 healthy and 4 patients with Parkinson's disease. On each 4 × 10 m recording we validate the estimated mean gait parameters over all strides against the motion capture (MoCap) reference using standard error metrics. In this manuscript the mean-absolute-error (MAE) of the mean gait speed within a gait test is presented as the main comparison metric. The evaluation is performed within a 5-fold cross-validation. For this challenge we compare the results of one pipeline (“Classic”) closely resampling the one presented in [56] and one pipeline (“Modern”) based on more recent algorithms presented in [24], [44], [45], [48], [49]. Note that both pipelines are not specifically optimized for this dataset, so the evaluation using cross-validation is not required. However, to ensure comparability with future algorithms, we still perform the evaluation this way. See Appendix 2, (Supplementary material) for more details.

## III. Results and Discussion

### A. Example Benchmarks

#### 1) Example 1 – Stride Segmentation

**Fig. 4.**
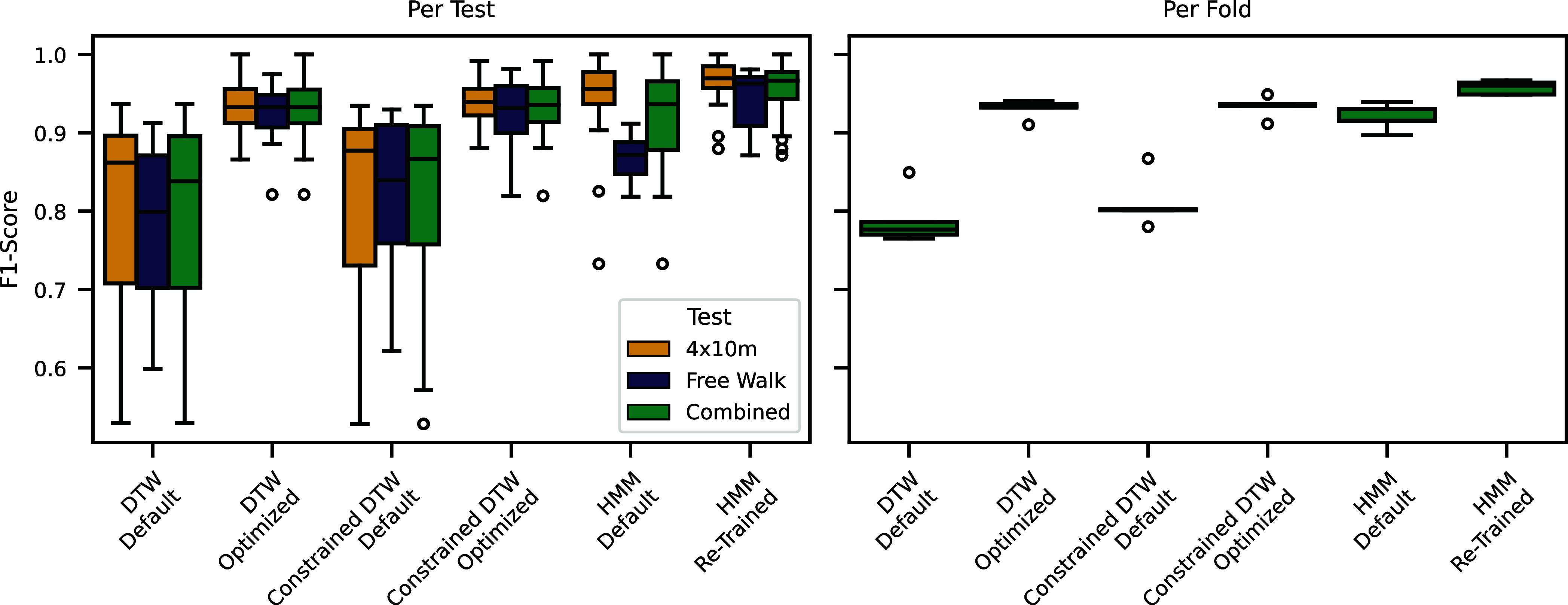
F1-score of the stride segmentation benchmark (Example 1) comparing all algorithms implemented in *gaitmap* in their default and optimized/re-trained version. In the left plot, each data point represents one gait test (either 4 × 10 m or 4-min walk). The data points are combined across all test-sets of the 5-fold cross-validation. In the right plot, each data point represents one test-set of the 5-fold cross-validation. All strides across all gait tests within each fold are grouped together before calculating the F1-score.

Comparing the different algorithms, the HMM appears to be the best overall approach (Fig. 4). It is able to provide good results, even without retraining the model for the specific dataset, and in the retrained version it shows the best performance overall. However, compared to the other algorithms, the HMM experiences a larger drop in performance going from the 4 × 10 m to the free-walking condition (Fig. 4, left). This highlights the need for validating algorithms under different conditions.

For all algorithms, optimizing the algorithmic parameters for the dataset increases the median F1-score and reduces the variation across data points. This underlines the importance of considering the hyperparameter optimization as part of an algorithmic approach. Fair comparison of algorithms is only possible if all algorithms are actively optimized for the specific task.

In their optimized/re-trained form, the algorithms implemented in *gaitmap* provide state-of-the-art performance with median F1-scores above 90%. However, the actual value differs between the per test and the per fold aggregation (Fig. 4). This shows that how exactly an error value is derived matters, and if they are not calculated absolutely identically, results can not be compared.

#### 2) Example 2 – Full Pipeline Validation

**Fig. 5.**
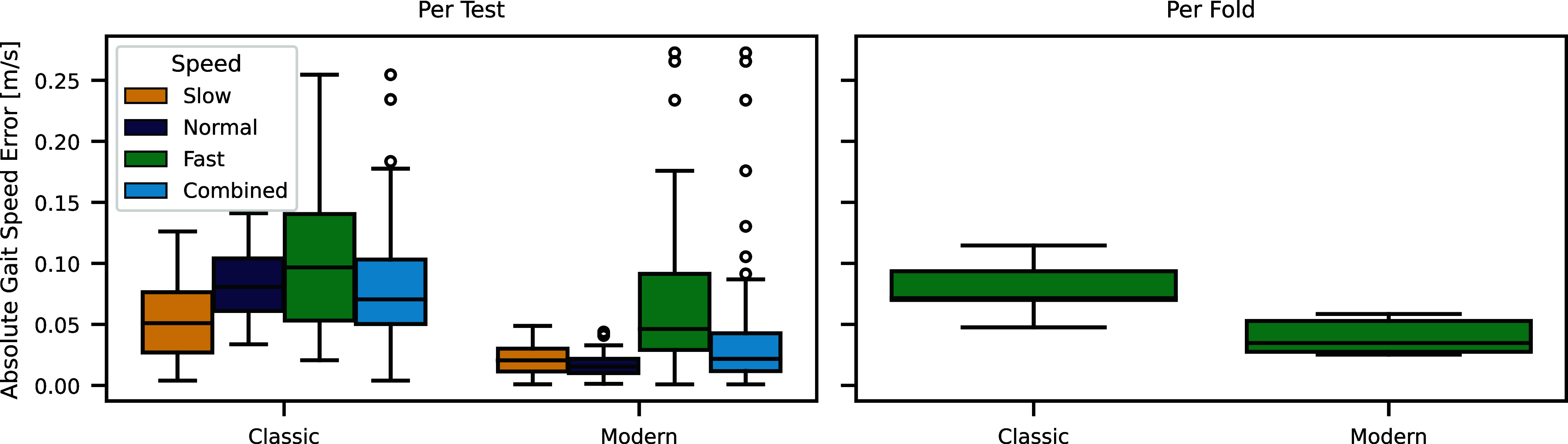
Absolute gait speed error of the full pipeline benchmark (Example 2) comparing the “Classic” and the “Modern” pipeline. In the left plot, each data point represents one 4 × 10 m gait test at different speeds. The data points are combined across all test-sets of the 5-fold cross-validation. Boxplots are provided for each gait speed and all data points combined. In the right plot, each data point represents one test-set of the 5-fold cross-validation. The absolute errors of the individual tests are averaged over each fold.

The “Modern” pipeline outperforms the “Classic” pipeline independent of the aggregation method and speed condition (Fig. 5). However, the speed dependency shows that combining the error to a single value (per fold aggregation) is not sufficient to fully understand the performance of the algorithms. Both pipelines show a speed dependency of the error, but the “Modern” pipeline has additional outliers at higher gait speeds, which are not present in the “Classic” pipeline. This indicates the need for in-depth analysis of individual data points to fully understand potential shortcomings of an algorithm.

The results from just these two example benchmarks show that a complete and fair comparison of algorithms requires full understanding of the comparison pipeline, access to algorithms' source code for optimization, and the ability to look at algorithm results in multiple ways and at multiple aggregation levels to understand the strengths and weaknesses of specific approaches. All of that is only possible, if data and code are shared fully and openly in an accessible way.

### B. Using and Extending the Ecosystem

One of the core aims of the presented ecosystem is to provide direct value to as many communities as possible. Hence, we split our software ecosystem into multiple independent open-source software packages with an increasing level of specificity. Ideally, this results in a larger user base of the more general packages, increasing the chance of building stable and sustainable communities around them. For example, the *tpcp* package [33], which was developed as the foundation for all the other packages, has already been used in the fields of digital psychology [58] and is being used in further applications fields our research group.

The *gaitmap* library itself has been used internally for the past years and was the basis for the data analysis in at least nine papers [44], [55], [59], [60], [61], [62], [63], [64] and numerous student theses. Furthermore, the developed algorithm pipelines are used by project partners in industry and clinical research. Based on these experiences, we are confident that the presented ecosystem will be useful far beyond our research group. To make adoption as seamless as possible, we provide extensive documentation and tutorials and are open to provide support.

As all of our libraries are built with extensibility in mind, they can be adapted for individual needs. All core algorithms are sensor-agnostic and only require that the data can be standardized into the correct format. Custom algorithms, datasets, and challenges can be added without modifying the core libraries and the provided benchmarks can be run fully locally to compare custom proprietary algorithms with presented baselines. To accommodate cases, where publishing algorithms and data openly is note possible, we ensured that the full ecosystem can be used (from a technical and legal perspective) for research purposes without the need of publishing your own code. This makes it possible to build (internal) toolboxes and workflows on top of our work. For example, we successfully integrated the *gaitmap* algorithms into a custom plugin for the *MaD*-GUI [65] to allow clinicians at the University Hospital Erlangen to analyze data from routine clinical gait recordings.

In case researchers can contribute their work back to the community, they can do so by adding their algorithms, datasets, or challenges to the libraries of the *gaitmap* ecosystem. Alternatively, they can use the *gaitmap* libraries as dependencies for their own packages and publish them independently, while still benefiting from the standardization and tooling we provide.

Beyond that, we are confident that our work demonstrates many best practices for developing scientific code and Python packages, and can therefore be used as a template for other projects.

### C. Limitations

To ensure the quality of the presented libraries, we decided to narrow the scope of this initial release to foot-worn IMUs. Given the current directions of the field of human gait analysis, similar projects including hip-worn or wrist-worn IMUs could be of high interest for the community. For this, the presented ecosystem could be extended to include new sensor positions or could serve as a blueprint for independent projects in the future.

Similarly, we focused on algorithms that were already used in our research group. This does not represent the entire field and the provided algorithms will likely not be the best for all use cases. Regarding datasets, we were limited by our attempt to build a fully open system. Most of the largest and interesting datasets are not publicly available. In particular, the inclusion of home-monitoring/real-world datasets and datasets including diverse participants with pathological gait would increase the usefulness of the presented benchmarks. We plan to extend the list of included algorithms and datasets in the future, and we believe that the visibility of this project can motivate others to support us in this process. In particular, recent promising machine learning based methods [14], [66], [67], [68], [69] are not included in this initial release. One of the main challenges for any open source ecosystem is to maintain and update the packages long term. With a strong focus on good software practices, testing, and documentation, we provide the foundation for that. However, the only sustainable long term solution is to have an active community around the presented ecosystem to make it independent of individual developers and funding sources. Building such a community is a difficult task, and we do not know yet how to best achieve this.

Regarding all the presented limitations, we are looking forward to open discussions within the community to improve and extend the presented ecosystem, and hope that this publication can motivate more open collaboration on computational tools.

## IV. Conclusion

This manuscript and the published code libraries represent the most comprehensive attempt yet to create an open ecosystem for analyzing human gait using IMUs and benchmarking the associated algorithms. The *gaitmap* ecosystem supports the development, evaluation, and usage of IMU-based gait analysis algorithms at every stage. This empowers researchers to write less and simpler code, allowing them to concentrate on their primary research questions. Moreover, it decreases the perceived burden of creating reproducible and publishable research code, facilitating broader evaluations and comparisons of algorithms across diverse datasets.

For clinical researchers and application developers this publication provides access to high quality implementations of state-of-the-art algorithms, which can be used to perform data analysis and build end-user applications for clinical and sports applications, reducing the required expertise and effort to do so.

In the long term, we hope the *gaitmap* ecosystem can grow into a community supported and independent project, facilitating open and reproducible research in human gait analysis and beyond.

## Supplementary Materials

Supplementary materials

## Author Contributions

AK lead the development of the gaitmap ecosystem, developed many of the core algorithms, and wrote the initial draft of the manuscript. AK, MU, MO, NR, AI, RR, MZ, FK, and BE were involved in planning and structuring the core software packages and the associated literature research. AK, MU, MO, NR, AI, FK, HM, RS, DP, LH, AS, MZ provided significant contributions to package development, the included algorithms, and documentation writing. All authors contributed to the writing of the manuscript and approved the final version.

## Conflict of Interest

The authors declare no conflicts of interest.

## Supplementary Materials

The supplementary material provides code examples on how to use the gaitmap-challenges library (Appendix 1) and further details on how the example benchmarks were performed (Appendix 2).
