# Supplementary material for: Gaitmap—An Open Ecosystem for IMU-Based Human Gait Analysis and Algorithm Benchmarking
Source: IEEE Open J Eng Med Biol. 2024 Jan 22;5:163–72. doi: 10.1109/OJEMB.2024.3356791 (PMC10939318; doi:10.1109/OJEMB.2024.3356791)
Supplement: Supplementary materials [file supp1-3356791.pdf]

APPENDIX 1 - EXAMPLE CODE

Example code snippets on how to run a benchmark-challenge using one of the *gaitmap* algorithms. Note, that these code snippets are simplified and might require further context to fully understand. They should mainly serve as a reference for the general structure and amount of the code required to run a benchmark. We recommend to check out the full documentation of the related software packages for more information.

Listing 1: Minimal code required to run the *BarthDtw* algorithms on the *EgaitSegmentationValidation2014* challenge. The `challenge.run()` call, runs the challenge. All additional code is required to provide the correct algorithm interface and store the final results. Imports are ommitted for brevity.

```python

# Defining the algorithms steps as a pipeline class
# We expect a instance of a DTW implementation as input.
# This way we can change, which DTW implementation we want to use.
class Egait2014DtwBase(Pipeline):
    def __init__(self, dtw):
        self.dtw = dtw

    def run(self, datapoint):
        # We convert the coordinate system to comply with gaitmap definitions
        bf_data = convert_to_fbf(
            Challenge.get_imu_data(datapoint), left_like="l", right_like="r"
        )

        # We use the dtw instance to predict the stride borders
        self.stride_list_ = (
            self.dtw.clone()
            .segment(bf_data, sampling_rate_hz=datapoint.sampling_rate_hz)
            .stride_list_
        )
        return self


if __name__ == "__main__":
    # We load some global configuration (e.g. dataset paths, n_cores for parallelization)
    config = set_config()
    # We set up the predefined challenge from the gaitmap-challenges package
    # It gets an instance of the corresponging dataset class
    challenge = Challenge(dataset=ChallengeDataset())
    # We run the challenge with the pipeline class we defined above
    # This will run a cross-validation and store the results in the challenge object
    # Because our pipeline is not a trainable algorithm, we wrap it in "DummyOptimize"
    # to use the pipeline with cross-validation.
    # This will simply skip the optimization step.
    challenge.run(
        DummyOptimize(
            pipeline=Egait2014DtwBase(dtw=BarthDtw()),
        )
    )
    # Finally we save the results as JSON.
    # The localtion is defined by our global configuration.
    save_run(
        challenge=challenge,
        entry_name=("gaitmap", "barth_dtw", "default"),
        custom_metadata=metadata,
    )
```

Listing 2: Additional code required to run the *BarthDtw* algorithms **with** parameter optimization using *Optuna* on the *EgaitSegmentationValidation2014* challenge. The used `Egait2014DtwBase` class is identical to the one in the previous example. The `challenge.run()` call, runs the challenge. All additional code is required to provide the correct algorithm interface and store the final results. Imports are ommitted for brevity.

```

# For Optuna we need to define a search space as a function
def optuna_search_space(trial):
    # We indicate that we want to search for optimal parameters of the
    # max_cost and the data columns used for the template
    # Because the "dtw" object is a "nested" object in the pipeline,
    # (see preiviouse example) we need to use "__" to indicate the nesting.
    trial.suggest_float("dtw__max_cost", 2.0, 3.5)
    trial.suggest_categorical(
        "dtw__template__use_cols",
        ['("gyr_ml",␣"gyr_si",␣"gyr_pa")', '("gyr_ml",␣"gyr_si")', '("gyr_ml",)'],
    )

# Then we need to setup the study parameters.
# We do that in a function that gets a random seed as input, to make sure
# that we get different initializations for each worker in the context of
# parallelization.
# Our search class will call this function for each worker with a different seed.
def get_study_params(seed):
    # We use a TPESampler, but every optuna sampler will work
    sampler = TPESampler(seed=seed)
    return {"direction": "maximize", "sampler": sampler}


if __name__ == "__main__":
    # We load some global configuration (e.g. dataset paths, n_cores for parallelization)
    config = set_config()
    # We set up the predefined challenge from the gaitmap-challenges package
    # It gets an instance of the corresponing dataset class
    challenge = Challenge(dataset=ChallengeDataset())
    # We run the challenge with the pipeline class we defined above.
    # This will run a cross-validation and store the results in the challenge object.
    # As we want to optimize the parameters of the pipeline, we wrap it in "OptunaSearch".
    # We use the functions defined above to define the search space and study parameters.
    # The cross-validation will run the parameter optimization for each fold separately on the
    # train data and then test the best parameters on the test data.
    challenge.run(
        OptunaSearch(
            pipeline=Egait2014DtwBase(dtw=BarthDtw()),
            get_study_params=get_study_params,
            scoring=challenge.get_scorer(),
            score_name="per_sample__f1_score",
            create_search_space=optuna_search_space,
            return_optimized=True,
            n_trials=100,
            eval_str_paras=["dtw__template__use_cols"],
        )
    )
    # Finally we save the results as JSON.
    # The localtion is defined by our global configuration.
    save_run(
        challenge=challenge,
        entry_name=("gaitmap", "barth_dtw", "optimized"),
        custom_metadata=metadata,
    )
```

APPENDIX 2 - DETAILS ON EXAMPLE BENCHMARKS

Example 1 – Stride Segmentation

This section provides supplementary information to the information already provided in the main text.

The actual implementation of the benchmark can be found at the links below:

- The challenge:
  https://github.com/mad-lab-fau/gaitmap-bench/blob/main/gaitmap-challenges/gaitmap_challenges/stride_segmentation/egait_segmentation_validation_2014.py
- Challenge entries:
  https://github.com/mad-lab-fau/gaitmap-bench/tree/main/entries/gaitmap_algos/gaitmap_algos/stride_segmentation (in the respective subfolders)
- Public results:
  https://gaitmap-bench.readthedocs.io/en/latest/challenges/Stride%20Segmentation/EgaitSegmentationValidation2014

The validation for this challenge is done within a 5-fold cross-validation. The splits are stratified by test and cohort, so that all train and test split contain approximately the same proportion of healthy controls and patients and the same proportion of 4x10m and free walk tests. As each participant only performed one of the tests, the train and test splits contain disjoint sets of participants.

Using the train fold of each split, the optimal parameters (see below) for each algorithm are determined. For the two DTW based methods, we optimized parameters using a Tree of Parzen Estimators (TPE) algorithm with 100 iterations implemented by the Optuna library. As part of this optimization, the optimal sensor axis and detection threshold are determined. For the `ConstrainedBarthDtw` algorithm, we also optimized the maximum template stretch factor, which limits the maximum stride duration that can be detected. We selected the parameters resulting in the highest F1-score over the train fold of each split. For the HMM algorithm, we did not optimize hyperparameters, to reduce the computational cost of the benchmark, but we retrained the model. Using the optimal parameters/retrained models we then evaluate the algorithms on the test fold of each split to obtain the “optimized” / “re-trained” results. For the “default” results, we did not perform any optimization and used the default parameters provided by *gaitmap*. For the HMM this uses a model trained on the FallriskPD dataset presented in Roth et al. (2021).

The final metrics were calculated in two ways. The “per-test” results are calculated by calculating the F1-score for each test individually. The boxplots for these results are generated by taking the data points from all test folds together. The “per-fold” results are calculated by first determining the True Positive, False Positives and False Negatives on a stride level. These counts are then combined across all tests within a fold and the F1-score is calculated. This means each stride is given equal weighting, regardless of the test it was performed in. Which of the aggregation methods is more appropriate to rank the algorithms and interpret the results depends on the use case.

Example 2 – Full Pipeline Validation

This section provides supplementary information to the information already provided in the main text.

The actual implementation of the benchmark can be found at the links below:

- The challenge:
  https://github.com/mad-lab-fau/gaitmap-bench/blob/main/gaitmap-challenges/gaitmap_challenges/full_pipeline/kluge_2017.py
- Challenge entries:
  https://github.com/mad-lab-fau/gaitmap-bench/tree/main/entries/gaitmap_algos/gaitmap_algos/full_pipeline (in the respective subfolders)
- Public results:
  https://gaitmap-bench.readthedocs.io/en/latest/challenges/Full%20Pipeline/Kluge2017

Like the other challenges, the validation for this challenge is done within a 5-fold cross-validation. For this dataset, each participant performed either 3 or 6 repetitions of the 4x10m test at different speed. To ensure that the train and test splits contain disjoint sets of participants, we performed a group-splits by participant that was further stratified by the patient label, to ensure that approximately the same distribution of healthy controls and patients is present in the train and test splits. As we did not perform any automated optimization for the pipelines we compared in the manuscript, the 5-fold cross-validation was not strictly necessary to create the results presented here. However, obtaining the results in this way allows for comparison with other algorithms with optimization steps in the future.

We did not perform any optimization here to reduce the computational cost of the benchmark. The combined pipeline has a multitude of parameters that could be optimized, and attempting to tune all of them would have been too costly. However, we set the parameters of the stride segmentation methods to the values that were obtained as optimal in the stride segmentation challenge.

The two algorithm pipelines that we compared both consisted of a different stride segmentation, event detection, and trajectory extraction step (see the actual implementation for the concrete steps). Afterwards, the same parameter extraction was used to obtain the spatial-temporal gait parameters per stride. As a final step, we aggregated the stride parameters to obtain a single value per test. Because, the motion capture reference only observed the center part of the tests and the turns of the 4x10m test were explicitly outside the capture volume, we also excluded all turning strides before aggregations of the algorithm results. This was done by removing all strides with a turning angle of 20 or more degrees. The running angle was estimated based on the calculated orientation of the foot at the start and end of the stride. The aggregation of the remaining strides was performed by averaging all parameters across strides.
